# Allergic Rhinitis and Ocular Allergy: A Correlative Study of Serum IgE, Tear Histamine, and Peripheral Eosinophils

**DOI:** 10.22336/rjo.2025.85

**Published:** 2025

**Authors:** Akash Chaudhary, Jung Bahadur Singh, Deependra Kumar Sinha, Ankita Singh, Ajit Kumar Singh, Ikjot Singh, Niloy Pathak

**Affiliations:** 1 ENT Department, Military Hospital Bathinda, Punjab, India; 2 Anaesthesia Department, Armed Forces Medical Services, India; 3 Military Hospital Bathinda, Punjab, India; 4 Ophthalmology Department, Military Hospital Bathinda, Punjab, India; 5 Anaesthesia Department, Military Hospital Bathinda, Punjab, India; 6 Pathology Department, Military Hospital Bathinda, Punjab, India

**Keywords:** allergic rhinitis, ocular allergy, serum IgE, tear histamine, eosinophils, visual analogue scale, AR = Allergic Rhinitis, OA = Ocular Allergy, IgE = Immunoglobulin E, VAS = Visual Analog Scale, ELISA = Enzyme-Linked Immunosorbent Assay, CBC = Complete Blood Count, PBS = Phosphate-Buffered Saline, ARIA = Allergic Rhinitis and its Impact on Asthma guidelines

## Abstract

**Background:**

Ocular allergies (OA) and allergic rhinitis (AR) commonly coexist, exacerbating patient symptoms and lowering quality of life. Dual diagnosis is still neglected despite this overlap.

**Objective:**

Assess peripheral eosinophil counts, tear histamine levels, and serum immunoglobulin E (IgE) in AR patients with and without ocular allergies, and correlate these with the intensity of visual symptoms.

**Methods:**

200 patients from a tertiary hospital were recruited for this prospective study: 100 with AR alone and 100 with AR+OA. Venous blood was used to measure eosinophils and serum IgE. Schirmer strips were used to measure tear histamine. A visual analogue scale (VAS) was used to score visual symptoms.

**Results:**

Patients with both AR and OA had significantly elevated serum IgE (230 ± 58 IU/mL), tear histamine (15.4 ± 4.2 ng/mL), and eosinophils (7.8 ± 2.1%) compared to AR-only patients (p<0.001). VAS scores were higher in the AR+OA group (7.1 ± 1.6 vs. 1.3 ± 0.6).

**Discussion:**

The findings demonstrate that combined AR and OA represent a more severe allergic phenotype, characterized by heightened systemic and local allergic responses. Elevated tear histamine strongly correlated with symptom severity, supporting its use as a practical, non-invasive marker of ocular involvement. The clear biomarker differences between groups highlight the need for routine ocular evaluation in AR patients to avoid underdiagnosis and to guide targeted therapy.

**Conclusion:**

Serum IgE, tear histamine, and eosinophils are elevated in AR+OA patients and may be used for screening, diagnosis, and monitoring of coexisting ocular allergy.

## Introduction

Allergic rhinitis (AR) is a chronic inflammatory condition that depends on IgE responses in the upper respiratory passages. The global prevalence of allergic rhinitis is increasing due to changes in lifestyle and environmental factors. The standard clinical presentation of allergic rhinitis includes sneezing, nasal congestion, rhinorrhea, and itching. Although ocular allergy (OA) affects 70% of patients with allergic rhinitis (AR) [**1-4**], its underdiagnosis remains common, as OA encompasses conditions such as allergic conjunctivitis and vernal or atopic keratoconjunctivitis. The common mucosal immune system connecting the nose and eyes is a critical factor in their simultaneous manifestation. The allergic response begins with contact with an allergen, which triggers sensitization and the release of IgE from mast cells. The immune response produces histamine, leukotrienes, and eosinophil chemoattractants as a result of this biological process [**5-8**]. The mediators trigger typical allergic reactions, including eye itching, redness, and tearing, and cause photophobia, nasal blockage, and sneezing with a runny nose. The standard practice in Ear, Nose, and Throat (ENT) assessments fails to consider eye-related symptoms. Diagnostic accuracy and treatment planning could be improved by objective biomarkers that detect both systemic and local inflammation. Serum IgE serves as an indicator of systemic allergic load, while ocular tear histamine indicates local mast cell activation in ocular tissue. In contrast, some authors report that eosinophils indicate active inflammation [**9-12**]. The investigation aimed to measure these biomarkers in Indian patients who now exhibit more severe allergic conditions due to urbanization and pollution. Our research aimed to measure these specific markers in AR patients, both with and without OA, and then compare these measurements with patients’ reported symptom severity [**13**].

## Methods

### Study design

This prospective, observational study was conducted at a single tertiary care hospital in Western India over 9 months, from June 2024 to February 2025. We received approval from the institutional ethics review board, and all participants were provided with written informed consent forms to sign.

### Patient selection

The study included patients aged 10-50 years who presented with clinical signs of allergic rhinitis (AR) at the ENT and ophthalmology outpatient departments. The diagnosis was made according to the ARIA guidelines [**14**].

Group A: 100 patients with AR only, and Group B: 100 patients with both AR and clinical features of ocular allergy.

To confirm ocular allergy, a detailed symptom history was taken, and a slit-lamp examination was done. Patients were excluded from the study if they had an autoimmune disease, any active infections, were pregnant, or had taken corticosteroids in the last four weeks.

### Clinical evaluation

Every patient involved in the study completed a symptom questionnaire. The visual symptoms we looked for included itching, watering, redness, and photophobia. We assessed the severity of these symptoms using a 10-point Visual Analog Scale (VAS), where 0 indicated no symptoms and 10 the maximum severity [**15**].

### Laboratory evaluation

- Serum IgE: We collected 5 mL of venous blood and processed the results using ELISA kits from Diagnostic Biotech Inc.
- Peripheral Eosinophils: These were obtained from an automated CBC analyser and identified with Leishman stain on the peripheral smear.
- Tear Histamine: We collected samples using Schirmer’s strips, which were placed in the lower conjunctival fornix for 5 minutes. The strips were then eluted in PBS and analysed via ELISA [**16**].

### Statistical analysis

The data was analysed using SPSS v25. Continuous variables were expressed as mean ± SD, and group differences were assessed using an independent t-test. Pearson correlation was applied to examine the relationship between tear histamine and VAS scores. A p-value of less than 0.05 was considered statistically significant [**17**].

## Results

**Graph 1:**
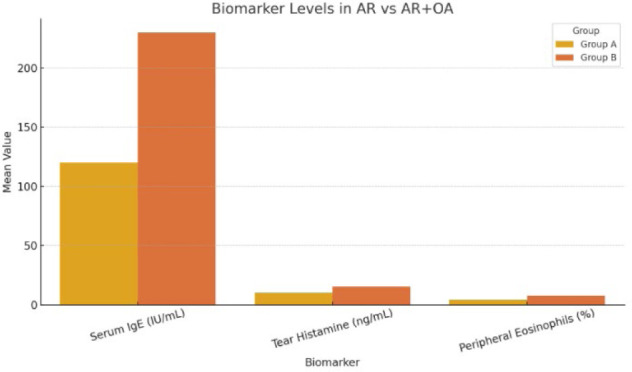
Bar chart – Biomarker comparison between groups

**Graph 2:**
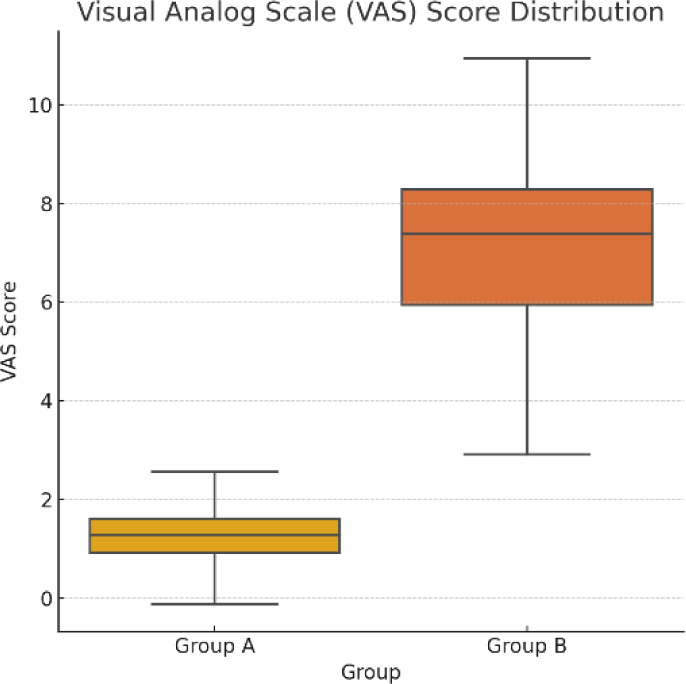
Box plot - VAS score distribution

The study involved 200 patients, of whom 55% were male, with a mean age of 28.6 ± 10.2 years. Of the sample, 47.5% had a family history of allergies, and a sizable portion (66.5%) resided in cities. Clinical symptoms showed that while ocular symptoms were unique to Group B, which included patients with both Ocular Allergy (OA) and Allergic Rhinitis (AR), nasal symptoms were shared by both groups (**Table 1, Graph 1**). Group A (AR only) had a mean VAS (Visual Analog Scale) score of 1.3 ± 0.6, while Group B (AR + OA) had a significantly higher mean score of 7.1 ± 1.6. This difference was statistically significant (p<0.001) (**Graph 2**).

**Table 1:** Biomarker comparison between groups

Parameter	Group A (AR only)	Group B (AR + OA)	p-value
Serum IgE (IU/mL)	120 ± 42	230 ± 58	<0.001
Tear Histamine (ng/mL)	9.8 ± 3.1	15.4 ± 4.2	<0.001
Peripheral Eosinophils (%)	4.2 ± 1.2	7.8 ± 2.1	<0.001
VAS Score	1.3 ± 0.6	7.1 ± 1.6	<0.001

In terms of laboratory findings, serum IgE, tear histamine, and peripheral eosinophils were all significantly higher in Group B compared to Group A. Specifically, the mean serum IgE level in Group A was 120 ± 42 IU/mL, whereas in Group B it was 230 ± 58 IU/mL (p<0.001). Tear histamine levels in Group A were 9.8 ± 3.1 ng/mL, while Group B had a mean of 15.4 ± 4.2 ng/mL (p<0.001). Similarly, the mean percentage of peripheral eosinophils in Group A was 4.2 ± 1.2%, while in Group B it was 7.8 ± 2.1% (p<0.001).

**Graph 3:**
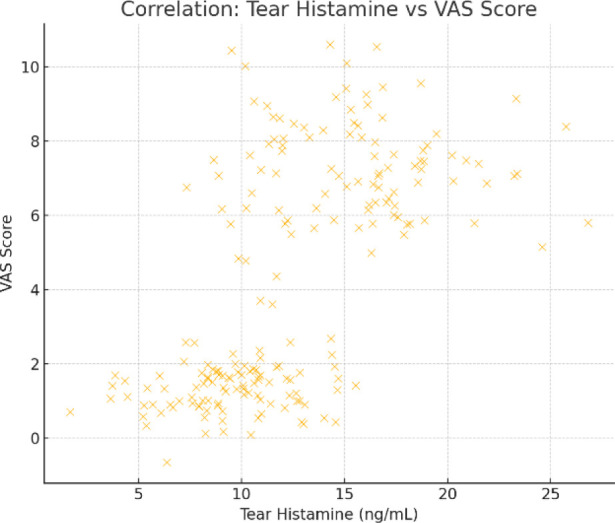
Scatter plot - Correlation between tear histamine and VAS score

Tear histamine levels and VAS scores were found to be strongly positively correlated, with a Pearson’s correlation coefficient of r = 0.68 (p<0.001) (**Graph 3**). This suggested that symptom severity, as measured by VAS scores, increased in tandem with tear histamine levels. These results highlighted the potential significance of tear histamine as a biomarker of symptom severity in patients with both OA and AR.

## Discussion

By demonstrating that patients with concurrent ocular symptoms had a heightened allergic response both locally and systemically, this study provided a significant contribution to our understanding of the relationship between ocular allergy (OA) and allergic rhinorrhea (AR). The systemic nature of the allergic response was highlighted by the higher serum IgE levels observed in patients with AR + OA compared with those with AR alone. This result was consistent with earlier research by Meltzer et al. [**18**] and Canonica et al. [**19**], which also found that patients with more severe allergic conditions had higher IgE levels. Elevated IgE indicated a strong immune response to allergens. Serum IgE did not reveal organ-specific involvement, especially in ocular tissues, even though it provided helpful information about systemic sensitization.

Despite being a good indicator of overall allergic sensitization, this limitation suggested that IgE might not adequately reflect the ocular component of the disease, particularly in patients with normal or only mildly elevated IgE levels who presented with isolated ocular symptoms. The study used tear histamine levels as a biomarker of localized ocular allergic inflammation to close this gap. This method offered a non-invasive way to measure mast cell activity in the eye itself using Schirmer’s strips.

In conjunction with serum IgE measurements, which showed systemic rather than organ-specific reactions, tear histamine provided a localized indicator of allergic response. The results were consistent with those of Spector et al. [**20-22**], who showed that ocular symptoms such as tearing and itching in allergic conjunctivitis were directly correlated with tear histamine levels. Group B, which had both AR and OA, had higher tear histamine levels, suggesting that their ocular inflammation was more severe than that in patients with AR alone. This implied that a more severe allergic reaction was caused by concurrent ocular involvement in AR, which might warrant targeted therapeutic interventions to address ocular symptoms.

The determination of eosinophil levels as an inflammatory marker was another strength of this study. The notion that chronic allergic inflammation was more severe in patients with both AR and OA was further supported by the marked increase in peripheral eosinophil levels in Group B compared with Group A. In allergic diseases, eosinophils play a crucial role in causing tissue damage and symptom persistence. This study extended the findings of Skoner et al. [**22**], who previously showed that eosinophil levels are a good measure of inflammatory activity in allergic respiratory diseases, to ocular allergies. Group B’s elevated eosinophils indicated that these patients were experiencing a more severe inflammatory response, especially in ocular tissues, which could contribute to symptom worsening.

The study’s findings were further strengthened using the Visual Analog Scale (VAS) to assess symptom severity. The study offered a more comprehensive understanding of the illness by establishing a correlation between subjective symptoms and objective biomarkers. Higher levels of serum IgE, tear histamine, and eosinophils were associated with Group B’s higher VAS scores, underscoring the importance of managing AR patients through both clinical and biochemical evaluations. This link between subjective symptoms and objective data improved the ability to measure symptom intensity and assess the efficacy of treatment approaches. Additionally, as discussed in earlier research, therapies could be tailored to biological markers and clinical presentation, enabling a more individualized approach to patient care [**23-25**].

Although the study’s approach had merit, a few points should be kept in mind. Its cross-sectional design was a significant drawback; it recorded data at a single point in time, making it impossible to analyse how biomarkers and clinical symptoms change over time, particularly in response to treatment. More details regarding the development of symptoms and biomarkers, as well as the long-term consequences of interventions, would be available from a longitudinal study. A control group of healthy individuals was also missing from the study, which would have strengthened comparisons of biomarkers between patients with AR (with and without OA) and non-allergic individuals.

The lack of longitudinal follow-up was another disadvantage, making it challenging to monitor the persistence of elevated biomarkers in response to management strategies or to evaluate the effect of treatment over time. A follow-up study would shed important light on the efficacy of various treatment modalities and whether biomarker changes are associated with better or worsening symptoms. Furthermore, possible confounding variables that could affect the study’s results were not considered, including exposure to environmental allergens and seasonal variations in allergic reactions. Future research would benefit from accounting for these factors to determine the specific effect of ocular involvement on the allergic response.

Despite these limitations, the 200-patient sample size was a noteworthy strength, ensuring more consistent and statistically significant results. A targeted examination of the specific biomarkers and symptoms associated with ocular involvement was made possible by the clear distinction between patients with AR only and those with both AR and OA. The results were reinforced by this unambiguous group classification, which also helped clarify the distinctions between the two patient groups. Furthermore, the study’s clinical relevance was enhanced by readily available biomarkers, such as serum IgE, tear histamine, and eosinophils, which may be used to evaluate and categorize patients with allergic diseases in standard clinical practice.

## Conclusion

As manifestations of a common allergic mechanism, AR and OA frequently coexist. Serum IgE, tear histamine, and peripheral eosinophil levels are markedly higher in patients with both disorders. In combination with VAS scores, these indicators can help guide diagnosis, assess severity, and track treatment effectiveness. For comprehensive allergy treatment, ENT and ophthalmology need to collaborate across multiple disciplines.
